# Hypoxic Preconditioning Increases Survival and Pro-Angiogenic Capacity of Human Cord Blood Mesenchymal Stromal Cells *In Vitro*

**DOI:** 10.1371/journal.pone.0138477

**Published:** 2015-09-18

**Authors:** Andreas Matthäus Bader, Kristin Klose, Karen Bieback, Dirk Korinth, Maria Schneider, Martina Seifert, Yeong-Hoon Choi, Andreas Kurtz, Volkmar Falk, Christof Stamm

**Affiliations:** 1 Berlin-Brandenburg Center for Regenerative Therapies, Charité—Universitätsmedizin Berlin, Berlin, Germany; 2 Institute of Transfusion Medicine and Immunology, Ruprecht-Karls University of Heidelberg, Mannheim, Germany; 3 Labor Berlin GmbH, Berlin, Germany; 4 Heart Center, University of Cologne, Cologne, Germany; 5 Deutsches Herzzentrum Berlin, Berlin, Germany; University of Torino, ITALY

## Abstract

Hypoxic preconditioning was shown to improve the therapeutic efficacy of bone marrow-derived multipotent mesenchymal stromal cells (MSCs) upon transplantation in ischemic tissue. Given the interest in clinical applications of umbilical cord blood-derived MSCs, we developed a specific hypoxic preconditioning protocol and investigated its anti-apoptotic and pro-angiogenic effects on cord blood MSCs undergoing simulated ischemia *in vitro* by subjecting them to hypoxia and nutrient deprivation with or without preceding hypoxic preconditioning. Cell number, metabolic activity, surface marker expression, chromosomal stability, apoptosis (caspases-3/7 activity) and necrosis were determined, and phosphorylation, mRNA expression and protein secretion of selected apoptosis and angiogenesis-regulating factors were quantified. Then, human umbilical vein endothelial cells (HUVEC) were subjected to simulated ischemia in co-culture with hypoxically preconditioned or naïve cord blood MSCs, and HUVEC proliferation was measured. Migration, proliferation and nitric oxide production of HUVECs were determined in presence of cord blood MSC-conditioned medium. Cord blood MSCs proved least sensitive to simulated ischemia when they were preconditioned for 24 h, while their basic behavior, immunophenotype and karyotype in culture remained unchanged. Here, “post-ischemic” cell number and metabolic activity were enhanced and caspase-3/7 activity and lactate dehydrogenase release were reduced as compared to non-preconditioned cells. Phosphorylation of AKT and BAD, mRNA expression of BCL-XL, BAG1 and VEGF, and VEGF protein secretion were higher in preconditioned cells. Hypoxically preconditioned cord blood MSCs enhanced HUVEC proliferation and migration, while nitric oxide production remained unchanged. We conclude that hypoxic preconditioning protects cord blood MSCs by activation of anti-apoptotic signaling mechanisms and enhances their angiogenic potential. Hence, hypoxic preconditioning might be a translationally relevant strategy to increase the tolerance of cord blood MSCs to ischemia and improve their therapeutic efficacy in clinical applications.

## Introduction

The potential of mesenchymal stromal cells (MSC) to treat ischemic diseases not amenable to other types of revascularization has been evaluated in clinical pilot trials, which showed encouraging results but also demonstrated the need for further refinement [1–3]. One of the factors that limit therapeutic efficacy is the poor survival of transplanted cells in the ischemic target tissue. To solve this problem, several strategies have been investigated, including preconditioning of the cell product by heat shock, oxidative stress or hypoxia [4]. Hypoxic preconditioning (HP) is known to protect somatic cells such as cardiomyocytes and endothelial cells from ischemic damage [5, 6], and a number of experimental studies have tested its applicability to MSC transplantation in animal models. In human bone marrow-derived MSCs, HP has been shown to increase their protective effects on cardiomyocytes, neurons and myocardial and hind limb ischemia [7–10]. Umbilical cord blood MSCs (CB-MSC) are believed to be particularly useful for tissue regeneration because their proliferative and functional capacity has not been hampered by age and disease, and they are free from acquired pathogens. Their extensive expansion capacity and low alloreactivity allow for the development of allogeneic “off-the-shelf” cell products, and they are increasingly cryopreserved at the time of birth for possible future autologous use. The therapeutic potential of CB-MSCs for the treatment of ischemic diseases has been demonstrated in clinical pilot trials [11, 12], and we have previously shown that CB-MSC-secreted factors protect cardiomyocytes and endothelial cells from ischemic damage [13, 14]. To further optimize the translational capacity of CB-MSCs, we have now developed a HP protocol that improves the ischemic tolerance of CB-MSCs and enhances their angiogenic profile in vitro.

## Material and Methods

### Cells and cell culture

Cryopreserved primary human CB-MSCs were provided by Karen Bieback, who isolated them from fresh umbilical cord blood as previously described [15]. Cord blood was obtained with written informed consent of the mother, according to the principles outlined in the Declaration of Helsinki and with approval of the Ethikkommission der Medizinischen Fakultät der Ruprecht-Karls-Universität Heidelberg and the Medizinische Ethikkommission II der Medizinischen Fakultät Mannheim der Ruprecht-Karls-Universität Heidelberg (Ref. 48/05 and 49/05 reconfirmed in 2009 and 2013). Cells were expanded in Dulbecco’s Modified Eagle Medium (DMEM), supplemented with 10% FBS, 100 U/ml penicillin, and 100 μg/ml streptomycin (“full medium”) at 37°C in a humid atmosphere of 21% O_2_ and 5% CO_2_ (all reagents from Life Technologies, Darmstadt, Germany). All experiments were performed on CB-MSCs in passage four. The phenotype of the used CB-MSCs as well as their ability to differentiate into non-hematopoietic cell types were repeatedly confirmed in previous experiments [13, 16]. Cryopreserved HUVECs were purchased from PromoCell, Heidelberg, Germany. Cells were expanded to the fifth passage and cultured in 0.1% gelatin-coated vessels in endothelial basal medium (EBM)-2 supplemented with endothelial growth medium (EGM)-2 growth factors, cytokines and supplements (Lonza, Basel, Switzerland) with 10% fetal bovine serum (FBS) (Life Technologies, Darmstadt, Germany) at 37°C and 5% CO_2_ in a humid atmosphere.

### Hypoxic preconditioning and simulated ischemia

For HP, CB-MSCs were subjected to an atmosphere of 1% O_2_ and 5% CO_2_, achieved by replacing O_2_ with N_2_ in an O_2_- and CO_2_-controlled multi gas incubator (Binder, Tuttlingen, Germany), while kept in full medium. For simulated ischemia, cells were subjected to the 1% O_2_, 5% CO_2_ atmosphere in glucose-free DMEM (Life Technologies, Darmstadt, Germany) without FBS. CB-MSCs incubated under normoxic conditions (21% O_2_) in full medium served as reference. The experimental protocol is depicted in S1 Fig.

### Flow cytometric immunophenotype analysis

CB-MSCs were harvested and washed with Dulbecco's Phosphate Buffered Saline containing 1% bovine serum albumin (DPBS/BSA). Cells were incubated for 15 min at 4°C in DPBS/BSA containing triple combinations of fluorochrome-conjugated antibodies: CD73-PE/CD90-APC/CD105-FITC and CD14-fluorescein/CD34-APC/CD45-VioBlue, respectively (S1 Table). Excess antibody was removed by washing with DPBS/BSA, and cells were analyzed in a FACSCanto II cytometer (BD Biosciences, Heidelberg, Germany). Data analysis was done using FlowJo software (FlowJo, LLC, Ashland, OR, USA).

### Karyotype analysis

Three hours prior to fixation, CB-MSCs were exposed to colcemid (0.02 mg/ml) to achieve metaphase arrest. After incubation in hypotonic KCl solution, the preparations were fixed three times with 3:1 methanol:glacial acetic acid and placed on water-rinsed glass slides. G-bands by trypsin using Wright’s stain (GTW) banding method was used for chromosome staining. The chromosome number was determined by microscopic analysis and metaphases were examined for the presence or absence of detectable structural rearrangements. Karyotypes were prepared from digitized images of twenty metaphases for each condition using Ikaros Karyotyping System (Metasystems, Altlußheim, Germany).

### Evaluation of CB-MSC damage

All experiments were performed in triplicate in CellCarrier 96-well plates (PerkinElmer, Rodgau-Jügesheim, Germany). Metabolic activity was determined using the Cell Counting Kit-8 (Dojindo EU, München, Germany). Cells were incubated for 4 h in medium containing water soluble tetrazolium (WST)-8. Absorbance at 450 nm, resulting from the conversion of WST-8 to WST-8 formazan by metabolically active cells, and at 650 nm (reference) was measured with a SpectraMax 340PC384 microplate reader (Molecular Devices, Biberach, Germany). Total cell number as well as nuclear shrinking and fragmentation were quantified using the high content imaging system Operetta^®^ with Harmony^®^ software (PerkinElmer, Waltham, MA, USA). Cells were fixed with 4% formaldehyde and nuclei were stained with Hoechst 33342 (Life Technologies, Darmstadt, Germany) and analyzed with the Operetta^®^ system. The nuclear fragmentation index (NFI) was defined as the coefficient of variation of nuclear stain fluorescence intensity. Caspase-3/7 activity was measured using the Caspase-Glo^®^ 3/7 Assay (Promega, Mannheim, Germany). Cells were washed with DPBS and then processed according to the manufacturer’s instructions. Luminescence was measured with a Mithras LB 940 multimode microplate reader (Berthold Technologies, Bad Wildbad, Germany) and normalized to the cell number, determined in simultaneously performed experiments with the Operetta^®^ system. Lactate dehydrogenase (LDH) release into the culture medium was quantified using the CytoTox-ONE^TM^ Homogeneous Membrane Integrity Assay (Promega, Mannheim, Germany) according to the manufacturer’s instructions. Fluorescence (ex 540 nm / em 590 nm) was measured with the Mithras LB 940 multimode microplate reader.

### Co-culture experiments

HUVECs were plated in the lower compartment of HTS Transwell-96 well plates with 1 μm pore size (Corning BV, Amsterdam, The Netherlands) and exposed to 24 h of simulated ischemia (1% O_2_ in glucose/FBS-free DMEM) with hypoxically preconditioned CB-MSCs (^HP^CB-MSC), ^HP^CB-MSC and anti-human VEGF antibody (S1 Table), naïve CB-MSCs, or cell-free medium in the upper compartment. 5-bromo-2-deoxyuridine (BrdU) uptake of HUVECs was determined with the Cell Proliferation ELISA, BrdU (Roche Diagnostics, Mannheim, Germany). BrdU was added to the cell culture medium during the final 4 h of the simulated ischemia co-culture period. Cells were then further processed according to the manufacturer’s instructions. Absorbance at 370 nm and 492 nm (reference) was measured with the SpectraMax 340PC384 microplate reader. The survival rate of HUVECs after 24 h of simulated ischemia was 63±1%, as determined in separate viability assays (S2 Fig).

### Conditioned medium experiments

CB-MSCs were seeded with a density of 10000 cells/cm². The cells were cultured for two days under normoxic standard conditions and were then exposed to 1% O_2_ for 24 h (HP) or left under normoxic standard conditions (non-HP). Then, both groups were washed with DPBS, covered with 0.15 ml/cm² glucose/FBS-free DMEM and subjected to the 1% O_2_ atmosphere for 24 h (simulated ischemia). Subsequently, conditioned medium was collected and filtered through Minisart^®^ NML syringe filters (Sartorius, Göttingen, Germany) to remove floating cells and debris. Concentrations of vascular endothelial growth factor (VEGF) and RANTES were determined with the QuantiGlo^®^ ELISA Human VEGF Immunoassay and the Quantikine^®^ Human RANTES Immunoassay, respectively (both R&D Systems, Wiesbaden, Germany). Monocyte chemotactic protein (MCP)-1 and interleukin (IL)-6 concentrations were determined with the Human MCP-1/CCL2 ELISA MAX™ Deluxe and the Human IL-6 ELISA MAX™ Deluxe (both Biolegend, Fell, Germany) according to the manufacturer’s instructions [17]. VEGF secretion was measured after three consecutive days of simulated ischemia. For migration experiments, HUVECs were serum starved for 3 h and plated onto gelatin-coated Nunc^TM^ cell culture inserts with 8 μm pore size in 12-well plates (Thermo Fischer, Langenselbold, Germany). CB-MSC-conditioned medium was placed in the bottom compartment of the wells and cells were incubated for 5 h. HUVECs that migrated to the bottom side of the membrane were fixed and stained with 0.1% crystal violet. Light microscopy images were taken from each membrane from the field of highest migration density and cells were manually counted. For determination of endothelial NO production, HUVECs were incubated in CB-MSC-conditioned EBM-2 for 16 h. The Nitric Oxide Synthase Detection System, Fluorometric (Sigma-Aldrich) was applied according to the manufacturer’s instructions. Fluorescence (ex 430 nm/em 535 nm) was measured with the Mithras LB 940 multimode microplate reader. Experiments were performed in triplicate.

### Gel electrophoresis and western blot analysis

Cells were washed with DPBS and lysed in sodium dodecyl sulfate (SDS) buffer containing Complete Mini Proteinase Inhibitor Cocktail Tablets and Phosstop Phosphatase Inhibitor Cocktail Tablets (both from Roche Diagnostics, Mannheim, Germany). Protein concentration was determined by bicinchoninic acid (BCA) protein assay (Thermo Scientific, Bonn, Germany), denatured protein (30 μg) was resolved in a 12% polyacrylamide SDS gel and transferred to a nitrocellulose membrane (Karl Roth, Karlsruhe, Germany). Membranes were blocked and incubated overnight at 4°C with primary antibodies against β-Actin, AKT, BCL2-associated agonist of cell death (BAD), phospho-AKT and phospho-BAD (S1 Table). Subsequently, membranes were incubated for 1 h with IRDye^®^ conjugated secondary antibodies (S1 Table) and blots were analyzed using the infrared imaging system and software Odyssey^®^ (Li-Cor Bioscience, Bad Homburg, Germany).

### Real time quantitative PCR (RT-qPCR)

Cells were washed with DPBS and RNA was purified using the RNeasy^®^ Mini Kit (Qiagen, Hilden, Germany). cDNA was synthesized from DNase-treated (Sigma-Aldrich, St. Louis, Missouri, USA) total RNA using the SuperScript^®^ III First-Strand Synthesis System for RT-PCR (Life Technologies, Darmstadt, Germany) with random hexamers as reaction primers. RT-qPCR was performed in a Mastercycler^®^ ep gradient S realplex2 (Eppendorf, Hamburg, Germany), using 2.5 ng template (7.5 ng for growth factors) in 25 μl reaction volume with 2 x Power SYBR^®^ Green PCR Master Mix (Life Technologies, Darmstadt, Germany) and gene specific primer pairs for β-actin, B-cell lymphoma-2 (BCL-2), B-cell lymphoma-extra-large (BCL-XL), BCL2-associated athanogene (BAG-1), Epidermal growth factor (EGF) and VEGF (S2 Table). Amplification conditions were as follows: 95°C for 10 min followed by 45 cycles consisting of 95°C for 15 s, the respective primer annealing temperature (S2 Table) for 30 s and 68°C for 60 s. Gene-of-interest expression (E) was calculated as E = PE ^(-Ct)^ (where PE is primer efficiency and Ct is the number of cycles at which the fluorescence exceeds the threshold) and normalized to β-actin expression. Primer efficiency was determined by means of calibration curves using the formula: PE = 10 ^(-1/slope)^. Measurements were performed in triplicate.

### Statistical analysis

Results are expressed as means ± SEM. The significance of intergroup differences was tested by one-way analysis of variance (ANOVA) with two-tailed Dunnett’s t-test. When only two groups were compared, a two-tailed Student’s t-test was performed. For intergroup comparisons of data obtained on different days, repeated-measures ANOVA was applied. IBM SPSS statistics 20 was used for data analysis. A P-value < 0.05 was considered significant.

## Results

### Hypoxic preconditioning protects CB-MSCs from ischemic damage

**Fig 1:**
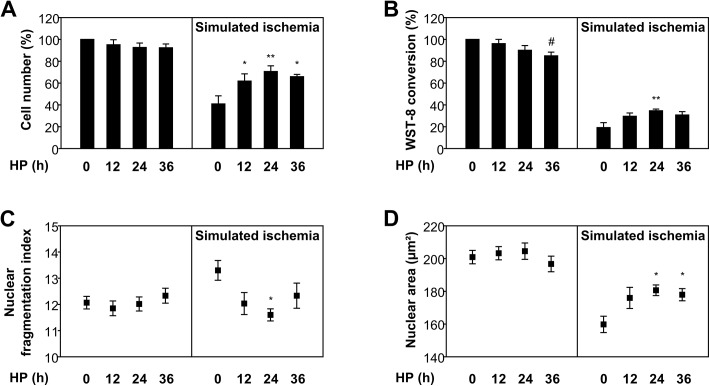
Reduced cell loss and damage of hypoxically preconditioned CB-MSCs. (A) Cell number, (B) WST-8 conversion, (C) nuclear fragmentation and (D) nuclear area after 0, 12, 24 or 36 h of hypoxic preconditioning (HP) followed by 24 h of cultivation under normoxic standard conditions (left panels) or simulated ischemia (SI) (n = 6). # P < 0.05 vs. non-HP/non-SI; * P < 0.05, ** P < 0.01 vs. non-HP/SI (ANOVA with Dunnett’s t-test). (A) and (B) expressed as the percentage of untreated cells (non-HP/non-SI).

In order to imitate ischemic conditions *in vitro*, CB-MSCs were deprived of glucose and serum while kept in a hypoxic atmosphere (1% O_2_) for 24 h. Prior to this “ischemic” challenge, cells were preconditioned by hypoxic incubation (1% O_2_) in full medium for 12, 24 or 36 h or kept under normoxic standard conditions (non-HP). “Ischemic” damage of CB-MSCs was reduced by HP and was lowest after a preconditioning period of 24 h. Here, the percentage of remaining viable cells was higher (71±5% of control vs. non-HP 41±7%, P < 0.01), WST-8 conversion rate was enhanced (35±2% of control vs. non-HP 19±5%, P < 0.01), the nuclear fragmentation index was lower (11.6±0.2 vs. non-HP 13.3±0.4, P < 0.05) and nuclear shrinking was attenuated (181±3 μm² nuclear area vs. non-HP 160±5 μm², P < 0.05) compared to non-preconditioned cells (Fig 1A–1D). Although some beneficial effects were also observed after shorter or longer preconditioning periods, the 24 h HP protocol proved most efficient and was used in all further experiments. Of note, a preconditioning period of 36 h also impaired baseline cell viability, with the WST-8 conversion rate of non-“ischemic” cells reduced to 85±3% of control (P < 0.05) (Fig 1B). “Ischemic” preconditioning, tested in preliminary experiments by shorter preceding phases of simulated ischemia was not protective, but rather amplified the detrimental effect of prolonged “ischemia” in CB-MSCs (S3 Fig).

### Hypoxic preconditioning does not alter the immunophenotype or karyotype of CB-MSCs

**Fig 2:**
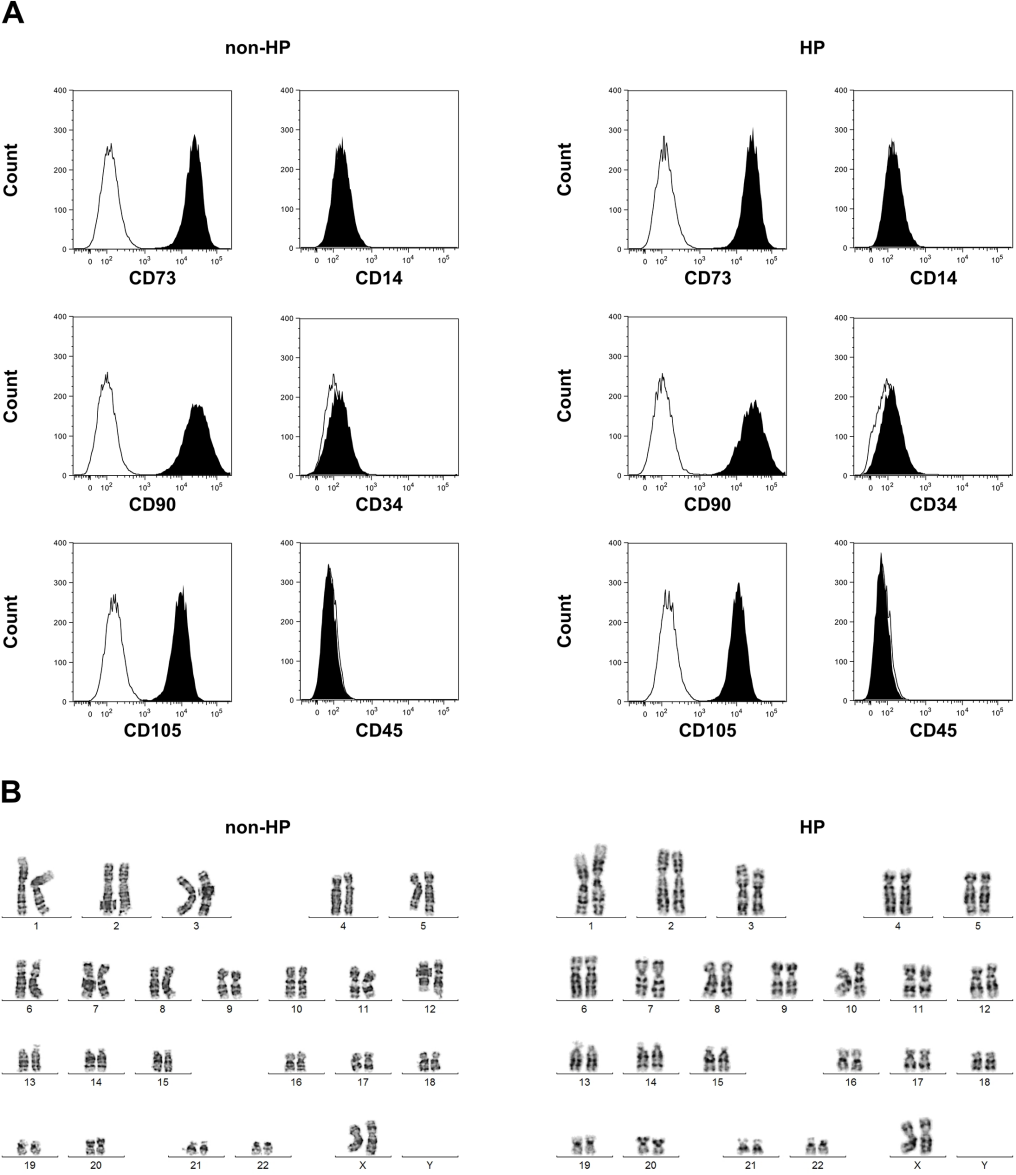
Immunophenotype and karyotype of hypoxically preconditioned CB-MSCs. (A) Expression of surface markers CD73, CD90, CD105, CD14, CD34 and CD45 by CB-MSCs prior (non-HP) and after hypoxic preconditioning (HP). (B) Representative karyograms of CB-MSCs prior an after HP.

In order to rule out unwanted cell modifications by HP treatment, immunophenotyping and karyotyping were performed prior and after HP. Expression of MSC positive markers CD73 (non-HP 99.7±0.1% vs. HP 99.7±0.1%, P = 0.7), CD90 (non-HP 99.5±0.03% vs. HP 99.6±0.1%, P = 0.7) and CD105 (non-HP 99.6±0.03% vs. HP 99.6±0.1%, P = 0.4) was unchanged by HP and so was expression of MSC negative markers CD14 (non-HP 0.12±0.02% vs. HP 0.16±0.01%, P = 0.1), CD34 (non-HP 0.25±0.04% vs. HP 0.32±0.05%, P = 0.3) and CD45 (non-HP 0.13±0.01% vs. HP 0.11±0.03%, P = 0.6). The surface marker expression pattern is shown in Fig 2A by representative histograms. HP also did not lead to chromosomal aberrations. After GTG banding, no clonal structural chromosomal changes were detectable at a level of 450 bands per haploid set of chromosomes, neither prior nor after HP. Representative karyograms are shown in Fig 2B.

### Hypoxic preconditioning activates anti-apoptotic mechanisms in “ischemic” CB-MSCs

**Fig 3:**
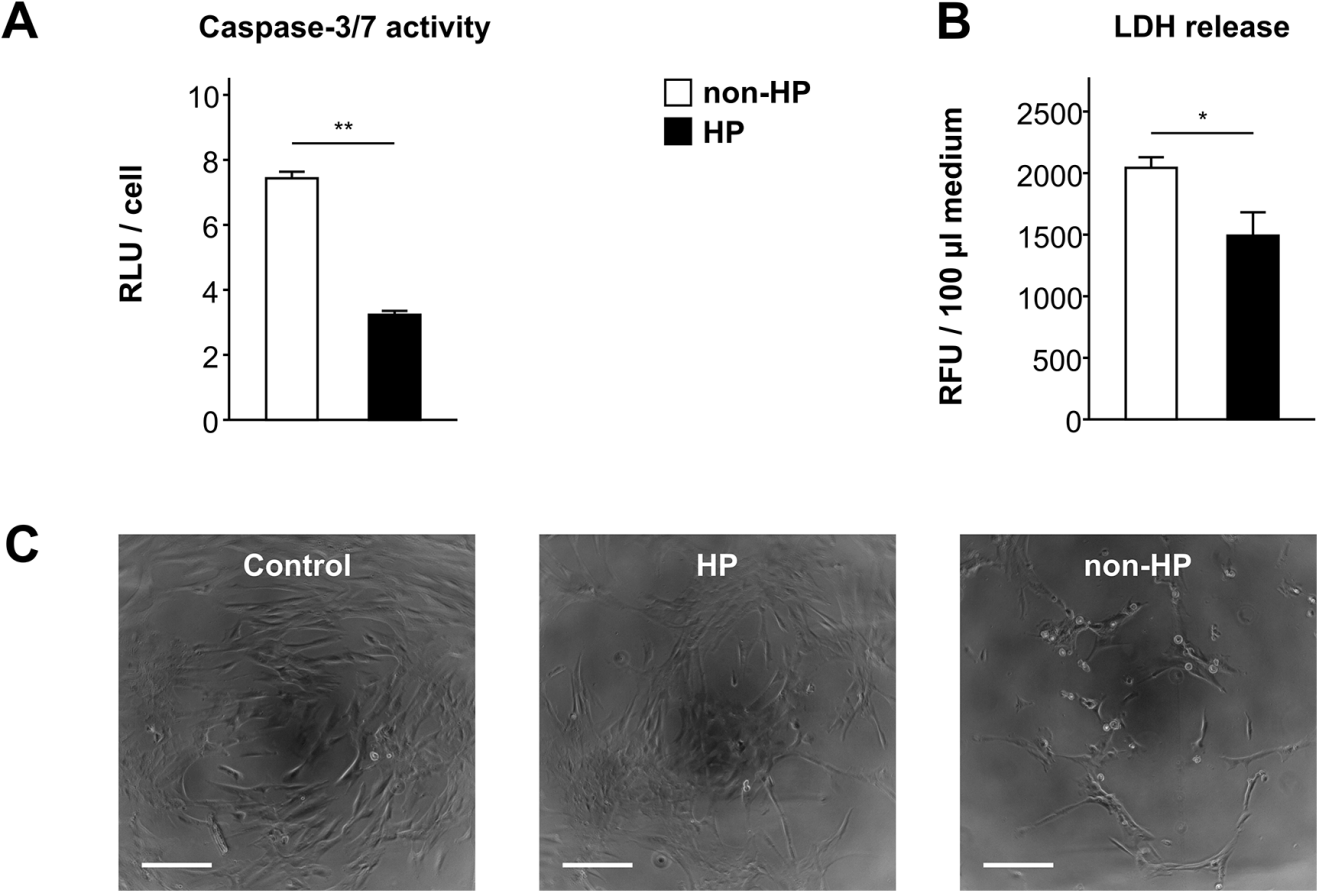
Reduced apoptosis of hypoxically preconditioned CB-MSCs. (A) Cellular caspase-3/7 activity (n = 3) and (B) LDH-release (n = 4) after 24 h of simulated ischemia with or without preceding hypoxic preconditioning (HP). * P < 0.05, ** P < 0.001 (Student’s t-test). (C) Representative microphotographs taken by light microscopy, magnification: 10x, scale bar = 200 μm. Control shows cells not exposed to hypoxic preconditioning/simulated ischemia.

**Fig 4:**
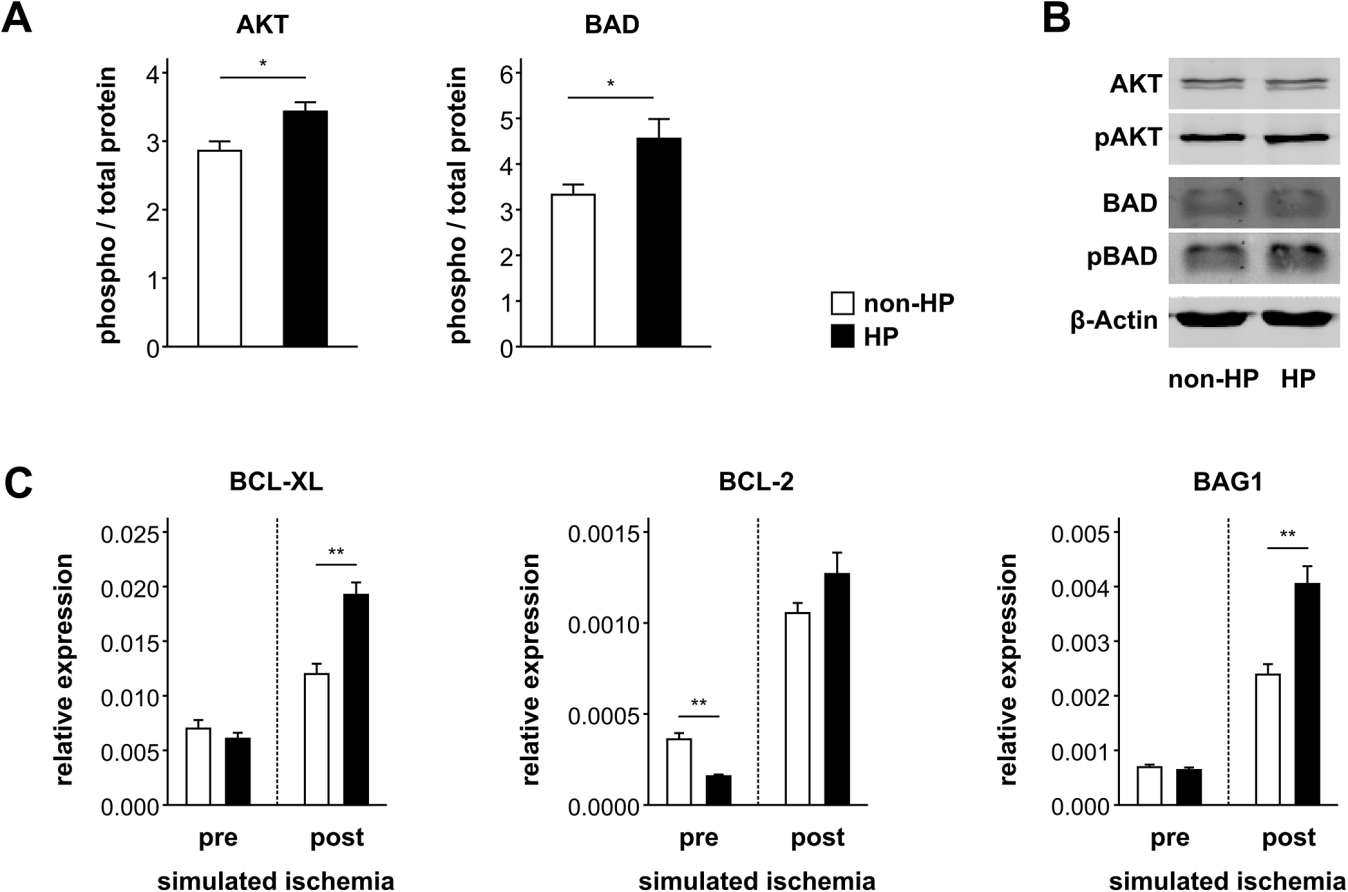
Protein phosphorylation and gene expression in hypoxically preconditioned CB-MSCs. (A) Phosphorylation of AKT and BAD after 24 h of simulated ischemia with or without preceding hypoxic preconditioning (HP) (n = 4). (B) Representative western blot images. (C) β-actin normalized mRNA expression of BCL-XL, BCL-2 and BAG1 prior and after 24 h of simulated ischemia with or without preceding hypoxic preconditioning (n = 6). * P < 0.05, ** P < 0.01 (Student’s t-test).

In hypoxically preconditioned CB-MSCs, caspase-3/7 activity after simulated ischemia was less than half of that measured in non-preconditioned cells (RLU/cell: 3.2±0.1 vs. non-HP 7.4±0.2, P < 0.001), and LDH release was also reduced (RFU/100 μl medium: 1492±190 vs. non-HP 2043±86; P < 0.05) (Fig 3A and 3B). Phosphorylation of the pro-survival kinase AKT was enhanced by HP (phospho-AKT/total AKT: 3.4±0.1 vs. non-HP 2.9±0.1, P < 0.05) (Fig 4A and 4B). In line with this, phosphorylation (i.e. inactivation) of the pro-apoptotic AKT target protein BAD was elevated (phospho-BAD/total BAD: 4.6±0.4 vs. non-HP 3.3±0.2, P < 0.05) (Fig 4A and 4B). Before the onset of simulated ischemia, mRNA expression of the anti-apoptotic factors BCL-XL and BAG1 was similar in preconditioned and non-preconditioned CB-MSCs, whereas BCL-2 expression was suppressed by HP (relative expression: 1.6±0.1×10^−4^ vs. non-HP 3.6±0.3×10^−4^; P < 0.01) (Fig 4C). Overall, mRNA expression of all three apoptosis-related genes increased during simulated ischemia. However, preconditioned cells showed significantly higher levels of BCL-XL (relative expression: 1.9±0.1×10^−2^ vs. non-HP 1.2±0.1×10^−2^; P < 0.01) and BAG1 (relative expression: 4.0±0.3×10^−3^ vs. non-HP 2.4±0.2×10^−3^; P < 0.01) as compared to control cells, and their initial loss of BCL-2 expression was fully compensated (relative expression: 1.3±0.1×10^−3^ vs. non-HP 1.1±0.1×10^−3^; P = 0.1) (Fig 4C).

### Hypoxic preconditioning enhances the pro-angiogenic effects of CB-MSCs

**Fig 5:**
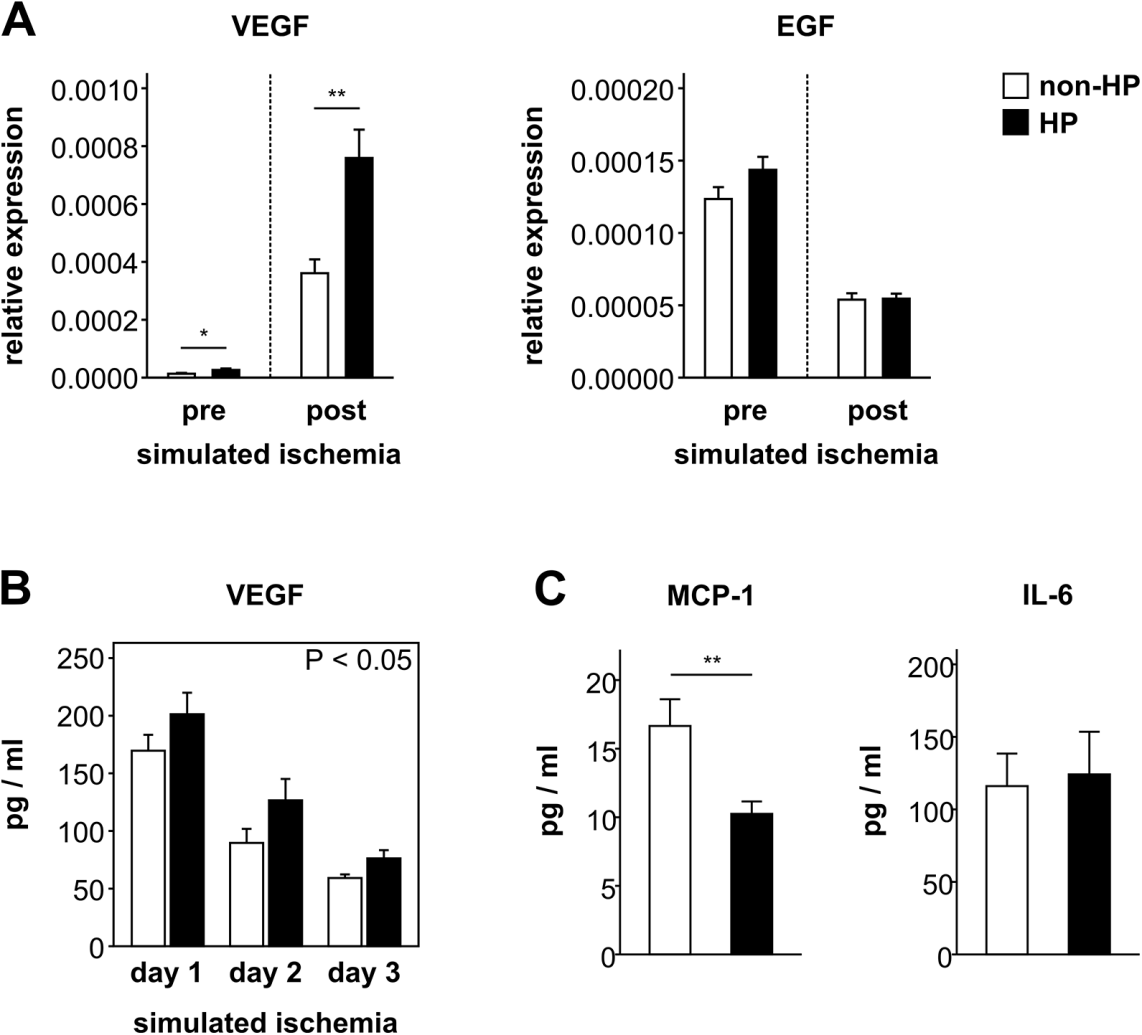
Growth factor and cytokine expression in hypoxically preconditioned CB-MSCs. (A) β-actin normalized mRNA expression of VEGF and EGF prior (n = 9) and after (n = 6) 24 h of simulated ischemia with or without preceding hypoxic preconditioning (HP). * P < 0.05, ** P < 0.01 (Student’s t-test). (B) VEGF protein secretion on three consecutive days of simulated ischemia with (n = 12) or without (n = 11) preceding hypoxic preconditioning. HP vs. non-HP: P < 0.05 (repeated measures ANOVA). (C) MCP-1 and IL-6 protein secretion during 24 h of simulated ischemia with (n = 12) or without (n = 11) preceding hypoxic preconditioning. ** P < 0.01 (Student’s t-test).

Prior to the onset of simulated ischemia, VEGF mRNA expression was already higher in preconditioned CB-MSCs than in non-preconditioned cells (relative expression: 2.7±0.5×10^−5^ vs. non-HP 1.4±0.3×10^−5^, P < 0.05) (Fig 5A). Although its expression markedly increased in both groups during simulated ischemia, the VEGF mRNA level was still twice as high in preconditioned cells (relative expression: 8±1×10^−4^ vs. non-HP 3.6±0.5×10^−4^, P < 0.01) (Fig 5A). VEGF protein secretion, measured on three consecutive days of simulated ischemia, was accordingly higher in hypoxically preconditioned CB-MSC (day 1: 201±19 pg/ml vs. non-HP 170±14 pg/ml; day 2: 127±19 pg/ml vs. non-HP 90±12 pg/ml; day 3: 76±7 pg/ml vs. non-HP 59±3 pg/ml, P < 0.05) (Fig 5B). EGF mRNA expression was not affected by HP and declined after simulated ischemia in both, preconditioned and non-preconditioned CB-MSCs (Fig 5A). The secretion of inflammatory cytokines during simulated ischemia was not triggered by HP. While IL-6 secretion was unchanged (124±29 pg/ml vs. non-HP 116±22 pg/ml, P = 0.8), hypoxically preconditioned CB-MSCs released even less MCP-1 (10±1 pg/ml vs. non-HP 17±2 pg/ml, P < 0.01) (Fig 5C). RANTES secretion was not detectable in any group.

**Fig 6:**
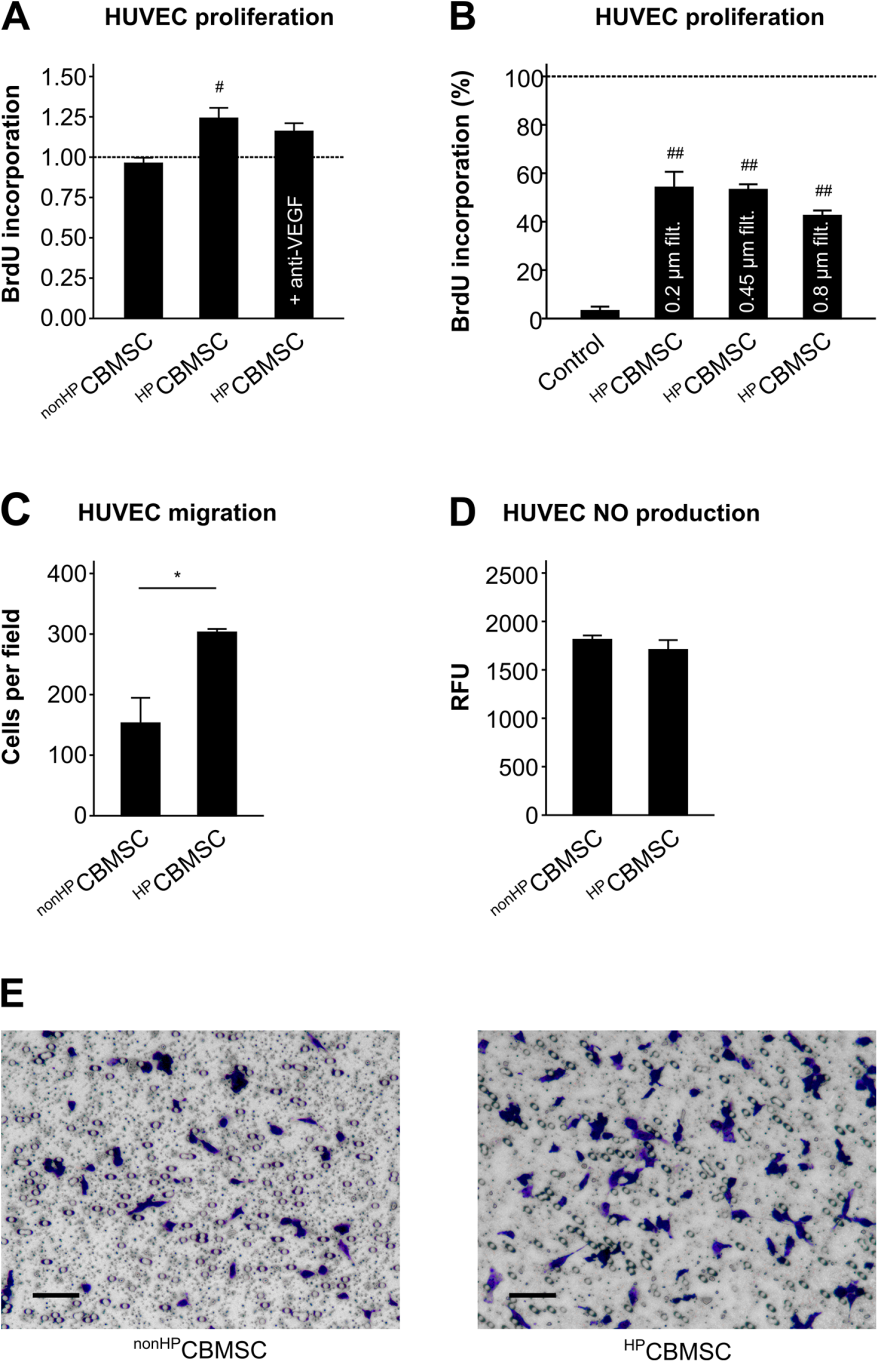
Pro-angiogenic effects of hypoxically preconditioned CB-MSCs (^HP^CB-MSC). (A) BrdU incorporation in HUVECs after 24 h of simulated ischemia in co-culture with ^HP^CB-MSCs or ^nonHP^CB-MSCs (n = 9) and with addition of anti-VEGF antibody (n = 3). Data expressed as multiples of HUVEC mono cell cultures without CB-MSCs (reference). ^#^ P < 0.01 vs. reference (ANOVA with Dunnett’s t-test). (B) BrdU incorporation in HUVECs after 6 h of simulated ischemia in 0.2 μm-, 0.45 μm- or 0.8 μm-filtered ^HP^CB-MSC-conditioned medium or in non-conditioned control medium (n = 3). Data expressed as percentage of HUVECs incubated in full medium at 21% O_2_ (reference). ^##^ P < 0.001 vs. reference (ANOVA with Dunnett’s t-test). (C) Transwell-migration (n = 3) and (D) NO synthesis (n = 4) of HUVECs in ^HP^CB-MSC or ^nonHP^CB-MSC-conditioned medium (0.2 μm-filtered). * P < 0.05 (Student’s t-test). (E) Representative microphotographs of HUVEC transwell-migration taken by light microscopy, magnification: 10x, scale bar = 100 μm.

We then studied the effect of CB-MSCs on endothelial cells in a simulated ischemia co-culture model. Proliferation (BrdU incorporation) of HUVECs under simulated ischemia was enhanced in co-culture with ^HP^CB-MSCs (proliferation index: 1.24±0.06, P < 0.01 vs. HUVEC mono cell cultures without CB-MSCs) but not with ^non-HP^CB-MSCs (proliferation index: 0.96±0.03) (Fig 6A). This mitogenic effect was partly neutralized in presence of an antibody against VEGF (Fig 6A) and was reproducible by ^HP^CB-MSC-conditioned medium filtered through 0.8 μm, 0.45 μm or 0.2 μm filters for removal of extracellular vesicles and debris beyond the respective pore size (all P < 0.001 vs. equivalent non-conditioned medium) (Fig 6B). ^HP^CB-MSC-conditioned medium also augmented the HUVEC migration capacity (migrated HUVECs per field: 303±6 vs. non-HP 153±42, P < 0.05) (Fig 6C and 6E), while NO production (RFU: 1706±102 vs. non-HP 1811±44, P = 0.4) remained unaffected (Fig 6D).

## Discussion

We have shown that HP increases the resistance of CB-MSCs to hypoxia and nutrient deprivation (“simulated ischemia”) *in vitro*, associated with phosphorylation of AKT and BAD as well as increased expression of BCL-XL and BAG1. In addition to a potentially better survival in ischemic tissue, such preconditioned CB-MSCs may support angiogenesis processes better than naïve CB-MSCs, because they secrete more VEGF and enhance the proliferation and migration capacity of endothelial cells.

Preconditioning of mesenchymal stromal cells has recently attracted attention as a means to increase the therapeutic efficacy of MSC products in the treatment of ischemic diseases [4]. The rationale is primarily that cell engraftment and retention proved very limited with unmodified cell products, and more advanced, i.e. genetic, modifications such as the overexpression of genes encoding for proteins involved in apoptosis signaling [18], are difficult to translate into clinical-grade protocols. The benefit of HP has mainly been demonstrated using cells of animal or human bone marrow origin, but little, if any information is available on cord blood-derived MSCs. Although CB-MSCs meet the minimal criteria defined for mesenchymal stromal cells, differences in metabolic profile and functional properties have been shown that underscore the need for unique CB-MSC handling strategies, as compared with bone marrow or adipose tissue MSCs [19]. Particularly the hypoxic environment in the fetal circulation gives reason to expect qualitative and/or quantitative differences to HP protocols, and tailored protocols are needed because with increasing numbers of cord blood units being banked for allogeneic or autologous use, CB-MSC applications are expected to gain in importance.

Moreover, in previous studies using bone marrow MSCs, a wide variety of preconditioning protocols have been used (Table 1) [7–10, 20–40]. It is important to distinguish between “hypoxic” preconditioning, i.e. the incubation in full medium at low ambient oxygen tension, and “ischemic” preconditioning, where cells are subject to hypoxia and glucose/serum deprivation. While the latter requires preconditioning periods of only 30 min to initiate cytoprotective effects [20–22], periods of up to several days are required to provoke purely hypoxia-induced pro-survival effects [23]. We found that the beneficial effects of HP are most pronounced after a pre-incubation period of 24 h, which is in line with most HP protocols described for human or animal bone marrow MSCs. Short-term ischemic preconditioning, however, proved to be detrimental to our human CB-MSCs and was hence not further investigated. For routine culture purposes, MSCs are known to tolerate or even favor lower oxygen tensions [41], and may react by enhanced proliferation and metabolic activity. As shown in Fig 1 (left panels), however, our HP protocol leaves the baseline CB-MSC properties unchanged and specifically modifies their reaction to simulated ischemia (right panels). As expected, our HP protocol does not induce chromosomal instability or alterations of the phenotype of the cells. Therefore, it might be added to existing CB-MSC cultivation protocols approved for clinical use, without having to re-confirm the stability of basic MSC properties.

**Table 1:** MSC hypoxic preconditioning strategies for treatment of ischemic cell/tissue injury.

Source	Method	Time	Experimental model	Reference
human—BM	hypoxia (1% O_2_)	48 h	*in vitro*; mouse medial hamstring muscles	Beegle et al., 2015 [23]
human—BM	hypoxia (1% O_2_) / reoxygenation (21% O_2_)	24 h / 5 d	*in vitro* coculture with rat cortical neurons	Kim et al., 2014 [8]
human—BM	hypoxia (0.5% O_2_)	24 h	*in vitro* coculture with murine cardiomyocytes	Hu et al., 2014 [7]
human—AT	hypoxia (1% O_2_)	24 h	rat acute kidney injury	Zhang et al., 2014 [28]
human—BM	hypoxia (1.5% O_2_)	7 d	swine myocardial infarction	Jaussaud et al., 2013 [9]
human—AT	hypoxia (0.5% O_2_)	24 h	mouse hindlimb ischemia	De Barros et al., 2013 [29]
human—BM	hypoxia (1–3% O_2_)	16 h	mouse hindlimb ischemia	Rosova et al., 2008 [10]
murine—BM	hypoxia (0.5% O_2_)	12 h	*in vitro*	Wang et al., 2014 [30]
murine—BM	hypoxia (0.5% O_2_)	12 h	*in vitro* coculture with mouse cardiac fibroblasts; mouse myocardial infarction	Chen et al., 2014 [31]
murine—BM	hypoxia (0.5% O_2_)	24 h	mouse myocardial infarction	Hu et al., 2014 [7]
murine—BM	hypoxia (1% O_2_)	24 h	*in vitro*; mouse hindlimb ischemia	Zhu et al., 2014 [26]
murine—BM	hypoxia (1% O_2_)	long term	mouse hindlimb ischemia	Huang et al., 2013 [32]
murine—BM	hypoxia (3% O_2_)	24 h	mouse acute kidney injury	Liu et al., 2012 [33]
murine—BM	hypoxia (0.5% O_2_)	24 h	rat myocardial infarction	Hu et al., 2011 [34]
murine—BM	hypoxia (1% O_2_)	36 h	mouse hindlimb ischemia	Leroux et al., 2010 [35]
murine—BM	hypoxia (0.5% O_2_) / reoxygenation (20% O_2_)	24 h / 2 h	rat myocardial infarction	Hu et al., 2008 [25]
murine—BM	anoxia / reoxygenation	4 h / 2 h	mouse myocardial infarction	Uemura et al., 2006 [36]
rat—BM	anoxia / reoxygenation with glucose/serum deprivation	30 min / 10 min (2x)	*in vitro*	Kim et al., 2012 [20]
rat—BM	anoxia / reoxygenation with glucose/serum deprivation	30 min / 10 min (2x)	*in vitro* coculture with rat cardiomyocytes; rat myocardial infarction	Kim et al., 2012 [21]
rat—BM	hypoxia (0.5% O_2_)	24 h	rat cerebral ischemia	Wei et al., 2012 [27]
rat—BM	hypoxia (1% O_2_) / reoxygenation (20% O_2_)	15 min / 30 min (2x)	*in vitro*	Peterson et al., 2010 [37]
rat—BM	hypoxia (0.5% O_2_)	24 h	*in vitro*	Chaco et al., 2010 [24]
rat—BM	anoxia / reoxygenation with glucose/serum deprivation	30 min / 10 min (2x)	rat myocardial infarction	Kim et al., 2009 [22]
rat—BM	anoxia	not given	rat myocardial infarction	Wang et al., 2009 [38]
rat—BM	anoxia with serum deprivation	3 h	rat myocardial infarction	He et al., 2009 [39]
rat—BM	hypoxia (8% O_2_) / reoxygenation (20% O_2_)	30 min / 30 min	*in vitro*	Wang et al., 2008 [40]

Listed are previously published studies that investigated effects of MSC hypoxic preconditioning in *in vitro* or *in vivo* models of (simulated) ischemic cell or tissue injury. If more than one preconditioning duration was tested, the most efficient is given. BM, bone marrow; AT, adipose tissue

The mechanisms that underlie HP-induced cytoprotection remain incompletely understood. Recently, Beegle et al. described a panel of HP-induced metabolic changes that were associated with better bone marrow MSC survival in muscle tissue [23], and Hu et al. highlighted the importance of leptin signaling in preconditioned bone marrow MSCs transplanted in the infracted myocardium [7]. We focused on signaling pathways involved in apoptotic cell death and/or angiogenesis processes and identified a typical pro-survival pattern. Phosphorylation of kinase AKT counteracts apoptosis and was previously shown to be triggered in MSCs by hypoxia [8, 10, 24, 42, 43], and we detected increased AKT phosphorylation in hypoxically preconditioned CB-MSCs after 24 h of simulated ischemia. Moreover, increased phosphorylation (i.e. inactivation) of the pro-apoptotic AKT target protein BAD was also persistent after 24 h of simulated ischemia. Anti-apoptotic BCL-2 family members are also induced in hypoxic MSCs [25]. In our experimental setting, HP-dependent induction of anti-apoptotic genes was delayed; immediately after hypoxic incubation, BCL-XL and BAG1 mRNA levels were still equal to normoxic control cells, while BCL-2 expression was even reduced. Only during the subsequent “ischemic” challenge the HP effects became apparent, with enhanced expression of BCL-XL and compensation of the initial loss of BCL-2. Simultaneously, expression of BAG1, an enhancer of the anti-apoptotic action of BCL-2, was elevated. Eventually, these pathways converge and inhibit the activity of effector caspases 3 and 7 in “ischemic” CB-MSCs. LDH release indicative of disruption of cell membrane integrity in necrosis was also reduced by HP, probably because in the absence of phagocytic cells, secondary necrosis is the ultimate fate of apoptotic cells [44].

HP has also been demonstrated to increase the pro-angiogenic properties of transplanted MSCs [7, 26, 27]. Preconditioned CB-MSCs enhanced the proliferation and migration of endothelial cells in an NO-independent manner. Previously we have shown, that CB-MSCs exposed to ischemia-like conditions secrete a variety of growth factors with angiogenic potency, amongst them VEGF and EGF [13], and now we tested whether their paracrine activity is further enhanced by HP. While mRNA expression of EGF did not respond, VEGF expression was augmented by the HP stimulus and this reinforcing effect was still present after a massive induction after 24 h of subsequent “ischemic” incubation. Given a higher number of surviving CB-MSCs with concomitantly higher VEGF gene expression, VEGF secretion was increased in preconditioned cells undergoing simulated ischemia. This secretory enhancement by HP was specific to VEGF and not seen for inflammatory cytokines IL-6, MCP-1 and RANTES. However, the effect of preconditioned CB-MSCs on endothelial cell proliferation was only partially mediated by VEGF. Beside soluble factors, extracellular vesicles (EV) have also been described to contribute to the effects of MSCs [45]. Here, we could show that EVs with a size greater than 0.2 μm were not involved in the mitogenic action of preconditioned CB-MSCs on endothelial cells.

The CB-MSCs we used were isolated from fresh cord blood, expanded, characterized, frozen, and thawed for further use as needed. In our hands, the success rate of MSC isolation is approximately 30%. We have been largely unsuccessful in isolating CB-MSCs from cryopreserved cord blood, although other groups reported more efficient but still incomplete MSC isolation from cryopreserved cord blood [46, 47]. It has been shown for various populations of MSCs that the basic cell characteristics (i.e. proliferation, differentiation, immunophenotype, immunosuppressive potential etc.) are not impaired by cryopreservation [48]. Therefore, we feel that the concept of using allogenic CB-MSCs with low allo-immunoreactivity, isolated from fresh CB and banked as cell products for future clinical use, has the highest translational potential. When autologous MSCs from neonatal tissues are the goal, umbilical cord tissue is a much more reliable cell source, but MSCs from Wharton’s jelly differ from CB-MSCs in several aspects and have not yet been studied as extensively as CB-MSCs.

## Summary

Taken together, we showed that our HP protocol increases the survival and potential pro-angiogenic capacity of human cord blood-derived MSCs in an ischemia-like environment, without influencing their baseline functional properties. Apoptosis-related signaling pathways are activated in the expected physiologic pattern, and this modus operandi may serve as a basis of future clinical preconditioning protocols tailored to the use of CB-MSC in regenerative medicine.

## Supporting Information

S1 Fig Experimental plan. (TIF)

S2 Fig HUVEC survival after 24 h of simulated ischemia. HUVECs were incubated for 24 h in DMEM with 1 g/L glucose and 10% FBS (“full medium”) at 21% O_2_ and in DMEM free of glucose and FBS at 1% O_2_, respectively. Subsequently, cells were incubated for 4 h at 21% O_2_ in full medium containing 3-(4,5-dimethylthiazol-2-yl)-5-(3-carboxymethoxyphenyl)-2-(4-sulfophenyl)-2H-tetrazolium (MTS) (Promega, Mannheim, Germany) and phenazine methosulfate (PMS) (Sigma-Aldrich, Taufkirchen, Germany). Absorbance at 490 nm and 650 nm (reference) was measured was with the SpectraMax 340PC384 microplate reader. * P < 0.01 (n = 3).
(TIF)

S3 Fig Adverse effect of “ischemic” preconditioning on CB-MSCs. (A) Cell number and (B) WST-8 conversion after 0, 30, 60 or 90 min of “ischemic” preconditioning (1% O_2_ in glucose/serum-free medium) followed by 24 h of cultivation under normoxic standard conditions (left panels) or 30 min “reperfusion” (21% O_2_ in full medium) and subsequent simulated ischemia (n = 1). Data expressed as the percentage of untreated cells.
(TIF)

S1 Table Antibodies. (DOCX)

S2 Table Oligonucleotides. (DOCX)
