# Supplementary figures and images for: Hypoxic Preconditioning Increases Survival and Pro-Angiogenic Capacity of Human Cord Blood Mesenchymal Stromal Cells In Vitro
Source: PLoS One. 2015 Sep 18;10(9):e0138477. doi: 10.1371/journal.pone.0138477 (PMC4575058; doi:10.1371/journal.pone.0138477)

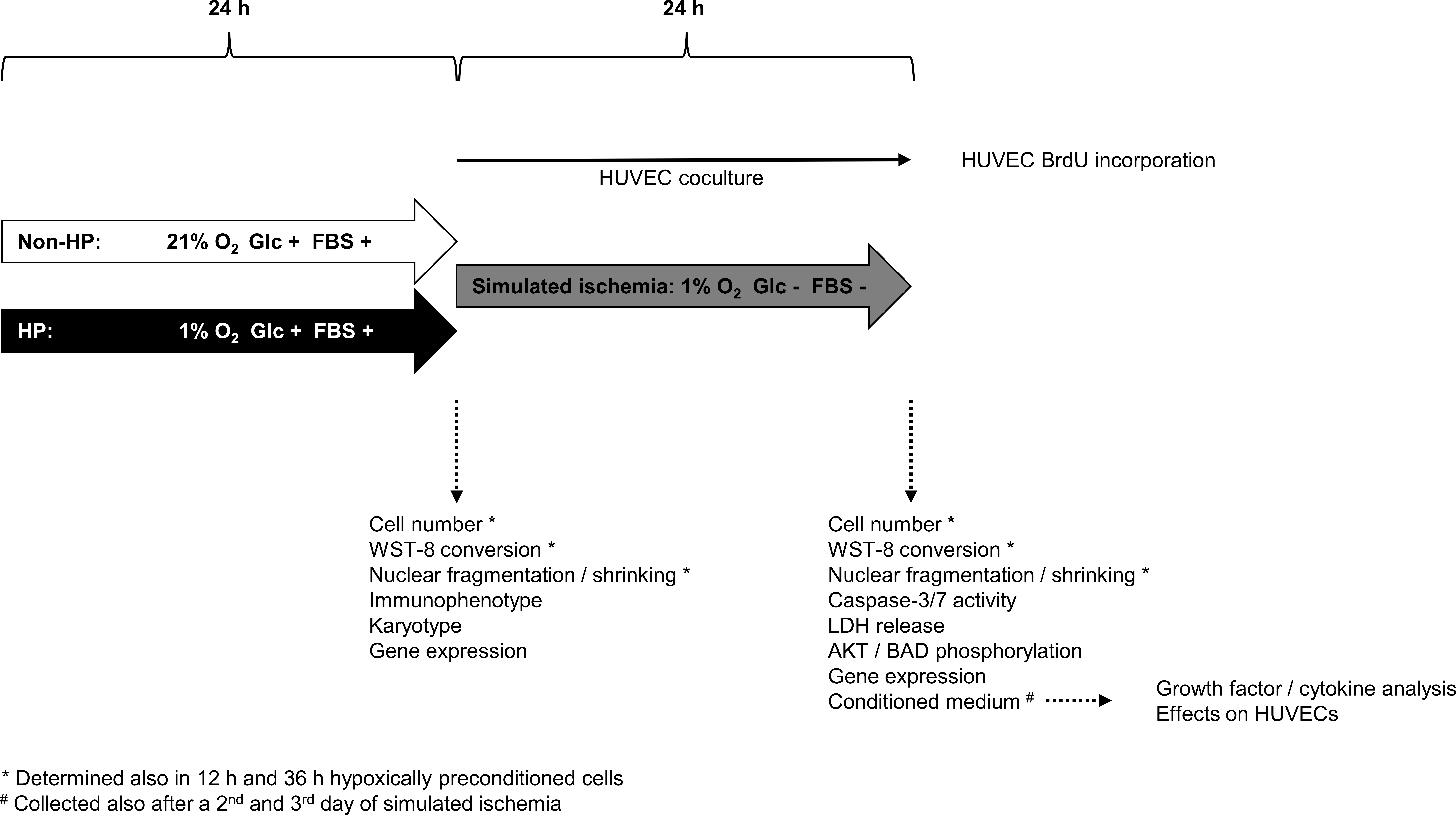

Supplement: S1 Fig — (TIF) [file pone.0138477.s001.tif]

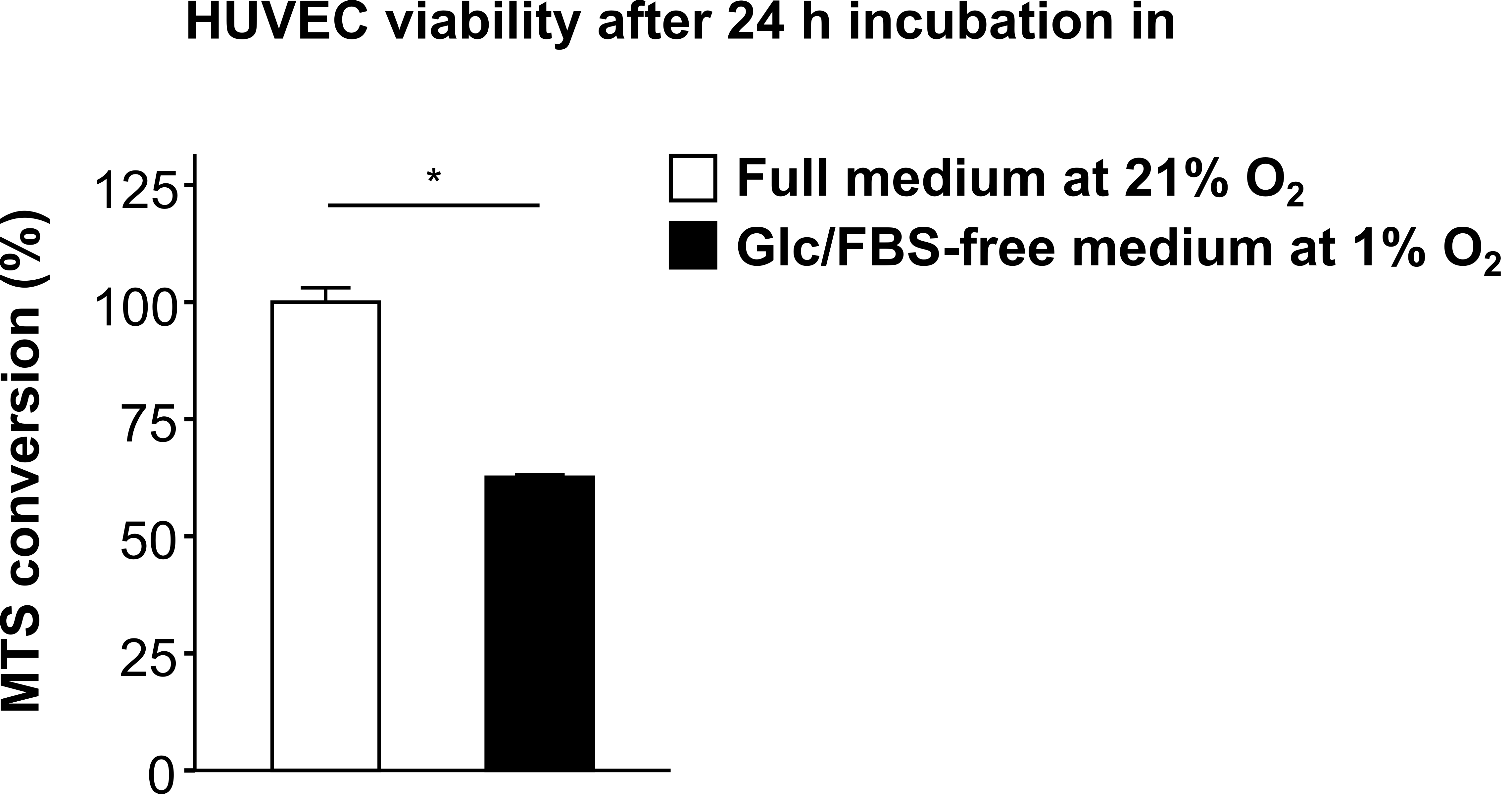

Supplement: S2 Fig — HUVECs were incubated for 24 h in DMEM with 1 g/L glucose and 10% FBS (“full medium”) at 21% O2 and in DMEM free of glucose and FBS at 1% O2, respectively. Subsequently, cells were incubated for 4 h at 21% O2 in full medium containing 3-(4,5-dimethylthiazol-2-yl)-5-(3-carboxymethoxyphenyl)-2-(4-sulfophenyl)-2H-tetrazolium (MTS) (Promega, Mannheim, Germany) and phenazine methosulfate (PMS) (Sigma-Aldrich, Taufkirchen, Germany). Absorbance at 490 nm and 650 nm (reference) was measured was with the SpectraMax 340PC384 microplate reader. * P < 0.01 (n = 3). (TIF) [file pone.0138477.s002.tif]

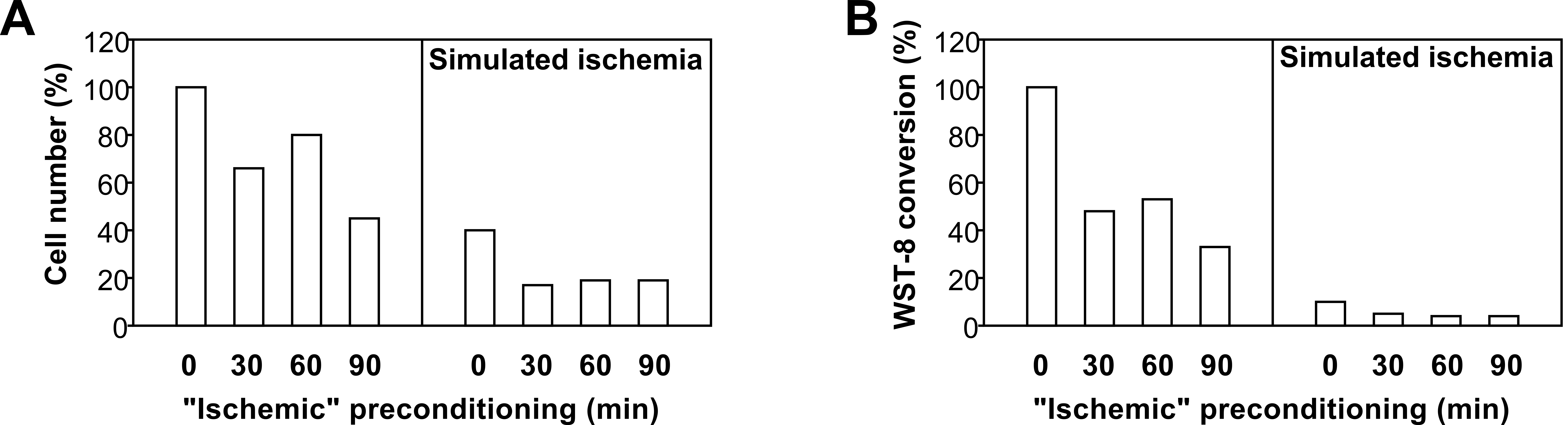

Supplement: S3 Fig — (A) Cell number and (B) WST-8 conversion after 0, 30, 60 or 90 min of “ischemic” preconditioning (1% O2 in glucose/serum-free medium) followed by 24 h of cultivation under normoxic standard conditions (left panels) or 30 min “reperfusion” (21% O2 in full medium) and subsequent simulated ischemia (n = 1). Data expressed as the percentage of untreated cells. (TIF) [file pone.0138477.s003.tif]
