# Supplementary material for: Hypoxic Preconditioning Increases Survival and Pro-Angiogenic Capacity of Human Cord Blood Mesenchymal Stromal Cells In Vitro
Source: PLoS One. 2015 Sep 18;10(9):e0138477. doi: 10.1371/journal.pone.0138477 (PMC4575058; doi:10.1371/journal.pone.0138477)
Supplement: S1 Table — (DOCX) [file pone.0138477.s004.docx]

| **Antibody name (clone)** | **Host species** |  | **Dilution** | **Catalogue #** | **Company** |
| --- | --- | --- | --- | --- | --- |
| Human VEGF Antibody (26503) | mouse | monoclonal | 1 µg/ml | MAB293 | R&D Systems, Abingdon, UK |
| Anti-beta Actin antibody (AC-15) | mouse | monoclonal | 1:30000 | Ab6276 | Abcam, Cambridge, UK |
| Purified Mouse Anti-Bad (48/Bad) | mouse | monoclonal | 1:250 | 610391 | BD Biosciences, Heidelberg, Germany |
| Akt (pan) (40D4) Mouse mAb | mouse | monoclonal | 1:2000 | 2920 | New England Biolabs, Frankfurt, Germany |
| Phospho-Akt (Ser473) (D9E) XP^®^ Rabbit mAb | rabbit | monoclonal | 1:2000 | 4060 | New England Biolabs, Frankfurt, Germany |
| Phospho-Bad (Ser136) (D25H8) Rabbit mAb | rabbit | monoclonal | 1:250 | 4366 | New England Biolabs, Frankfurt, Germany |
| IRDye^®^ 680LT Conjugated Goat (polyclonal) Anti-Mouse IgG (H+L), Highly Cross Absorbed | goat | polyclonal | 1:30000 | 926-68020 | Li-Cor Bioscience, Bad Homburg, Germany |
| IRDye^®^ 800CW Conjugated Goat (polyclonal) Anti-Rabbit IgG (H+L), Highly Cross Absorbed | goat | polyclonal | 1:15000 | 926-32211 | Li-Cor Bioscience, Bad Homburg, Germany |
| CD14 Antibody (MEM18) Fluorescein Labeled | mouse | monoclonal | 1:5 | GM-4092 | ADG Bio Research, Kaumberg, Austria |
| CD34-APC human (AC136) | mouse | monoclonal | 1:10 | 130-090-954 | Miltenyi Biotec, Bergisch Gladbach, Germany |
| CD45-VioBlue human (5B1) | mouse | monoclonal | 1:10 | 130-092-880 | Miltenyi Biotec, Bergisch Gladbach, Germany |
| PE anti-human CD73 (AD2) | mouse | monoclonal | 1:10 | 344004 | BioLegend, Fell, Germany |
| APC Conjugated Anti-human CD90 (5E10) | mouse | monoclonal | 1:5 | 17-0909-73 | eBioscience, Frankfurt am Main, Germany |
| Anti-CD105 antibody (FITC) (MEM-226) | mouse | monoclonal | 1:5 | ABIN93940 | antibodies-online, Aachen, Germany |
