# Supplementary material for: Hypoxic Preconditioning Increases Survival and Pro-Angiogenic Capacity of Human Cord Blood Mesenchymal Stromal Cells In Vitro
Source: PLoS One. 2015 Sep 18;10(9):e0138477. doi: 10.1371/journal.pone.0138477 (PMC4575058; doi:10.1371/journal.pone.0138477)
Supplement: S2 Table — (DOCX) [file pone.0138477.s005.docx]

| **Gene** | **Forward primer** | **Reverse primer** | **Concentration** | **Annealing temperature** |
| --- | --- | --- | --- | --- |
| β-Actin | 5’-CATGTACGTTGCTATCCAGGC-3’ | 5’-CTCCTTAATGTCACGCACGAT-3’ | 300 nM | 56°C |
| BCL-2 | 5’-GAACTGGGGGAGGATTGTGG-3’ | 5’-GCCGGTTCAGGTACTCAGTC-3’ | 150 nM | 60°C |
| BCL-XL | 5’-GAGCTGGTGGTTGACTTTCTC-3’ | 5’-TCCATCTCCGATTCAGTCCCT-3’ | 300 nM | 56°C |
| BAG-1 | 5’-TGAGAAGCACGACCTTCATGT-3’ | 5’-GGAACCCCTATGACCTCTTCA-3’ | 300 nM | 56°C |
| EGF | 5’- CAGGGAAGATGACCACCACT-3’ | 5’- TTCCCACCACTTCAGGTCTC-3’ | 500 nM | 58°C |
| VEGF | 5’-AGACACACCCACCCACATAC-3’ | 5’-TGCCAGAGTCTCTCATCTCC-3’ | 100 nM | 58 °C |
